# Resveratrol potentiates the *in vitro* and *in vivo* anti-tumoral effects of curcumin in head and neck carcinomas

**DOI:** 10.18632/oncotarget.2534

**Published:** 2014-09-26

**Authors:** Laura Masuelli, Enrica Di Stefano, Massimo Fantini, Rosanna Mattera, Monica Benvenuto, Laura Marzocchella, Pamela Sacchetti, Chiara Focaccetti, Roberta Bernardini, Ilaria Tresoldi, Valerio Izzi, Maurizio Mattei, Giovanni Vanni Frajese, Florigio Lista, Andrea Modesti, Roberto Bei

**Affiliations:** ^1^ Department of Experimental Medicine, University of Rome “Sapienza”, Rome, Italy; ^2^ Department of Clinical Sciences and Translational Medicine, University of Rome “Tor Vergata”, Rome, Italy; ^3^ STA, University of Rome “Tor Vergata”, Rome, Italy; ^4^ Dipartimento di Scienze Motorie, Umane e della Salute, Università di Roma, Foro Italico; ^5^ Centro Studi e Ricerche Sanità e Veterinaria Esercito, Rome, Italy

**Keywords:** polyphenols, head and neck cancer, curcumin, resveratrol

## Abstract

The survival rate of head and neck squamous cell carcinomas (HNSCC) patients has not considerably changed over the last two decades. Polyphenols inhibit the growth of cancer cells. We determined whether the combination of Resveratrol (RES) and Curcumin (CUR) enhanced their *in vitro* and *in vivo* antitumor activities on HNSCC cell lines compared to the single compounds. We provide evidence that RES potentiated the apoptotic effect and reduced the IC50 of CUR on HNSCC cell lines. The model of compounds interaction indicated the onset of an additive effect of the two compounds compared to the single treatment after decrease of their concentrations. RES+CUR compared to CUR increased the PARP-1 cleavage, the Bax/Bcl-2 ratio, the inhibition of ERK1 and ERK2 phosphorylation, and the expression of LC3 II simultaneously with the formation of autophagic vacuoles. RES and CUR induced cytoplasmic NF-κB accumulation. RES+CUR administrations were safe in BALB/c mice and reduced the growth of transplanted salivary gland cancer cells (SALTO) more efficiently than CUR. Overall, combinations of CUR and RES was more effective in inhibiting *in vivo* and *in vitro* cancer growth than the treatment with CUR. Additional studies will be needed to define the therapeutic potential of these compounds in combination.

## INTRODUCTION

The rate of head and neck squamous cell carcinomas (HNSCCs) is increasing worldwide, and despite advances in treatment, the survival rate of HNSCC patients has not considerably changed over the last two decades [1]. The development of HNSCC is multistep, progressing from precancerous lesions to malignant tumors [2].

Polyphenols constitute one of the most numerous and widely distributed groups of natural products in the plant kingdom [3]. Polyphenols can be employed to inhibit the growth of cancer cells due to their ability to modulate the activity of multiple targets involved in carcinogenesis through simultaneous direct interaction or modulation of gene expression [4, 5].

Curcumin (CUR) and Resveratrol (RES) are non-flavonoid polyphenols [3, 6]. CUR [l,7-bis-(4-hydroxy-3-methoxyphenyl)-l,6-heptadiene-3,5-dione], found in the spice turmeric, a product of the plant *Curcuma longa*, has been widely employed for centuries in Asia as a food additive as well as in cosmetic and herbal medicine. CUR is a pleiotropic molecule able to interact with a variety of molecular targets and signal transduction pathways and has been revealed to have antitumor, anti-inflammatory, antioxidant, immunomodulatory and antimicrobial activities in both rodents and humans [7-11]. Due to its ability to modulate the activity of multiple targets involved in carcinogenesis through direct interaction or modulation of gene expression, CUR is considered a “multifunctional drug” [7, 9-11]. However, CUR has poor absorption, biodistribution, metabolism, and bioavailability, which might hinder the *in vivo* effects of the compound [7]. Indeed, in a phase I clinical trial for patients with advanced colorectal cancer refractory to standard chemotherapies, the oral administration of 3.6 g of curcumin daily produced a plasma CUR level in the 10 nmol/L range after 1 hour [12]. RES (3,4′,5-trihydroxy-transstilbene), a polyphenol compound isolated from grapes, berries, plums, peanuts and pines, has several biological properties, including antioxidant, anti-inflammatory, anticancer and anti-aging activities [13-15]. Similar to CUR, RES may have partial biological activity due to poor absorption and first-pass metabolism [16, 17]. It has been reported that RES and CUR inhibit the growth of HNSCC cell lines when employed as single drugs [18-25]. Overall, the poor bioavailability of CUR and RES will affect the effective dose delivered to cancer cells. One way to counteract this drawback could be combination treatment with CUR plus RES, which can lead to more effective anti-tumoral effects than treatment using only one of the compounds. We previously demonstrated that RES enhanced CUR-induced sarcoma cell apoptosis [26].

The aim of this study was to determine whether the combination of RES and CUR resulted in an enhancement of their *in vitro* and *in vivo* antitumor activities on HNSCC cell lines compared to the single compounds. In addition, we explored the effect of these compounds and their interaction with signal transduction pathways involved in apoptosis and the growth of cancer cells.

## RESULTS

### Inhibition of human HNSCC cell survival by RES and CUR alone or in combination

**Figure 1:**
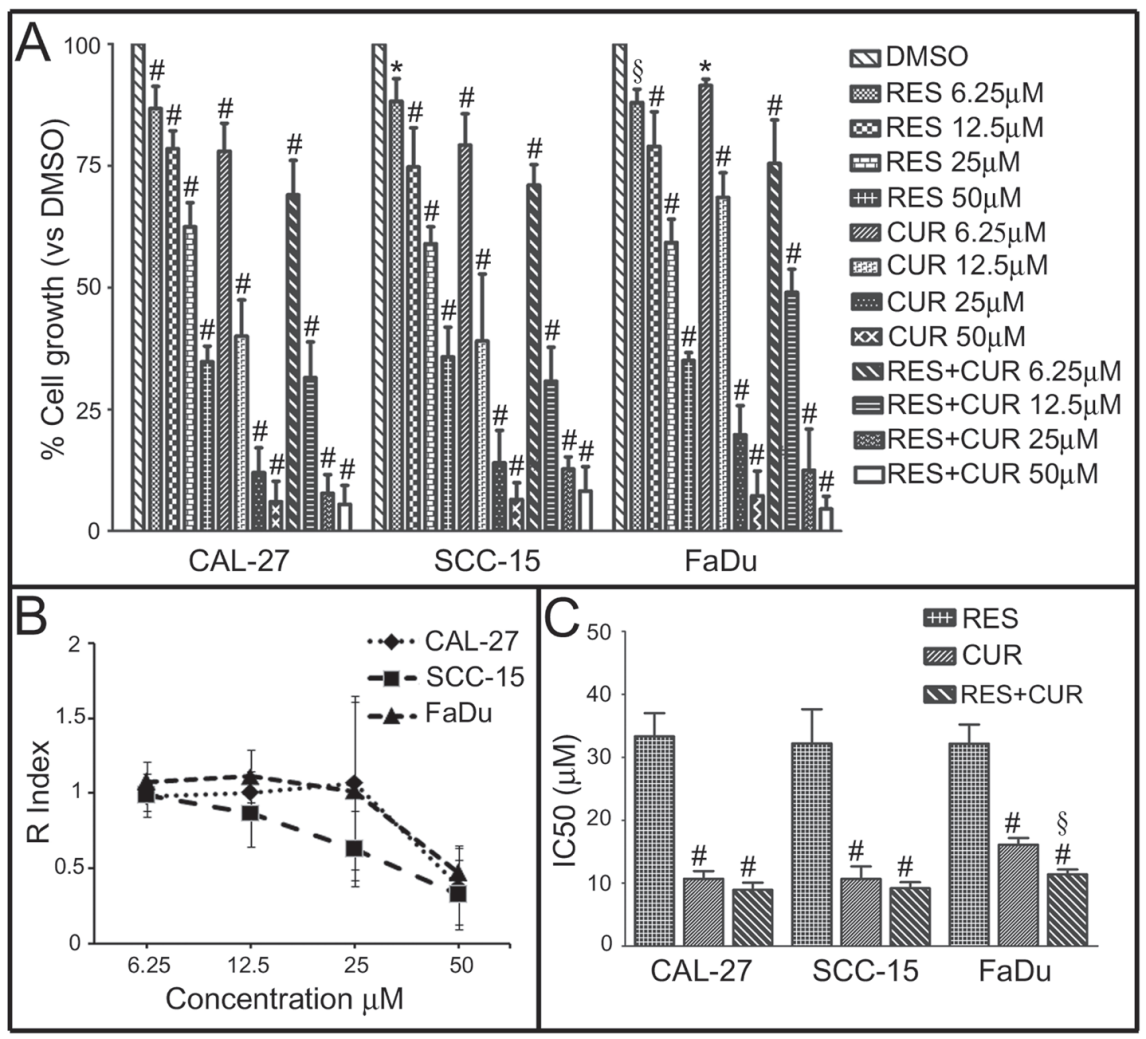
Effect of RES and CUR alone or in combination on HNSCC cell survival Panel A: Survival of tongue (CAL-27 and SCC-15) and pharynx (FaDu) cancer cells was assessed by the SRB assay after 48 hours of treatment with DMSO, RES or CUR alone or in equimolar combinations of the two compounds (RES+CUR). The results are reported as the mean ± SD values from three experiments performed in triplicate. #: p<0.001; §: p<0.01; *: p<0.05 vs cultures treated with DMSO. Panel B: Interaction between RES and CUR on the growth of HNSCC. The graph represents the KERN index (R) after treatment. R > 1 represents a synergistic effect, and R < 1 indicates that the effect of the combined treatment is less than additive. R=1 indicates that the effect is additive. Panel C: Inhibitory concentration of 50% with respect to cell growth (IC50) of CAL-27, SCC-15 and FaDu cells after treatment with CUR and RES alone or in combination. #: p< 0.001, CUR vs RES and RES+CUR vs RES; §: p<0.01, RES+CUR vs CUR.

The survival of tongue (CAL-27 and SCC-15) and pharynx (FaDu) cancer cells was evaluated by the SRB assay after exposure to increasing doses of RES and CUR alone or in combination (RES+CUR) or vehicle control (DMSO) for 48 hours. The effects of CUR and RES were dose-dependent and achieved statistical significance at all doses tested compared to vehicle control treatment (Figure 1, Panel A). However, CUR was the most effective compound in inhibiting cell survival. The effect obtained with equimolar combinations of RES+CUR was significantly higher than the effect of treatment with RES at all concentrations on CAL-27 (p<0.001), SCC-15 and FaDu cells (p<0.001 at 50-25-12.5 μM; p<0.01 at 6.25 μM for both cell lines) or CUR alone at 12.5-6.25 μM (CAL-27 and FaDu, p<0.05) or at 6.25 μM (SCC-15) (p<0.05) (Figure 1, Panel A).

The model of interaction between CUR and RES when used in combination was determined using the method of Kern (Figure 1, Panel B). Interaction between RES+CUR at the concentration of 50 μM indicates an R index of 0.38, 0.32 and 0.49 after treatment of CAL-27, SCC-15 and FADU cells, respectively, which indicates a less than additive effect. However, R increases in all cell lines when the concentrations of the compounds decreased, which indicates the onset of an additive effect of the two compounds with respect to the associated single treatment after decrease of their concentrations (Figure 1, Panel B).

The concentration of compounds that inhibits 50% of cell growth (IC50) was also determined. RES+CUR significantly reduced the IC50 compared to treatment with the single CUR treatment in FADU cells (Figure 1, Panel C).

### RES potentiates the apoptotic effect of CUR on human HNSCC lines

To determine the effects of the compounds alone or in combination on apoptosis and cell cycle distribution of HNSCC cells, a FACS analysis of DNA content was performed. The effects of the compounds were compared to each other and to DMSO (Table 1). RES resulted in a dose-dependent decrease in the percentage of G0/G1 cells (CAL-27: p<0.001 at 25 and 12.5 μM; SCC15: p<0.001 at 25 μM and p<0.01 at 12.5 μM; FaDu: p<0.001 at 25-6.25 μM) and an increased percentage of S phase cells (CAL-27 and SCC15: p<0.05 at 25 μM; FaDu: p<0.01 at 12.5 and 6.25 μM). RES did not change the apoptotic sub-G1 population. Conversely, CUR resulted in a marked, dose dependent increase of the percentage cells in the sub-G1 phase (CAL-27: p<0.001 at 25 μM; SCC15: p<0.001 at 25 and 12.5 μM; FaDu: p<0.05 at 25 μM) and in a decrease in the number of G0/G1 cells (CAL-27: p<0.001 at 25 μM; SCC15: p<0.01 at 25 and 12.5 μM; FaDu: p<0.001 at 25 and 12.5 μM and p<0.01 at 6.25 μM). As for the effects of the combined treatment, RES+CUR induced a significant, dose-dependent increase in the percentage of apoptotic, sub-G1 cells in all cell lines compared to either compound alone at a higher dose (Table 1). From the comparison of the apoptotic rates obtained with the RES+CUR treatment and the data obtained with CUR alone, it emerged that in CAL-27 cells, the combined treatment allowed a reduction of the dose of CUR required to achieve an apoptotic rate of 28% by 1.8 times. Similarly, the dose of CUR required to achieve an apoptotic rate of 58% and 25% could be reduced by 1.94 and 2.77 times through the combination of CUR and RES in SCC-15 and FaDu cells, respectively.

**Table 1:** Effects of RES and CUR alone or in combination on the cell cycle of cell lines derived from HNSCCs of the tongue (CAL-27, SCC-15) or pharynx (FaDu)

**CAL-27**		**SUB-G1**		**G0/G1**		**S**		**G2/M**	
		**Mean±SD^1^**	**p**	**Mean±SD**	**p**	**Mean±SD**	**p**	**Mean±SD**	**p**
	**DMSO**	1.2±0.4		54.9±4.4		15.8±1.3		27.9±3.1	
	**RES 6.25**	0.9±0.6		39.7±10.4		21.3±3.9		38.0±7.0	
	**RES 12.5**	1.4±0.9		25.3±11.2		23.8±5.4		49.4±7.6	
	**RES 25**	2.7±1		15.3±5		28.0±3.2		53.9±3.8	
	**CUR 6.25**	0.9±0.4		47.6±6.5		17.3±2.2		34.1±4.3	
	**CUR 12.5**	1.6±1.1		50.2±3.7		14.5±1,1		33.4±4.9	
	**CUR 25**	15.3±1.6		20.7±6.1		23.9±8		39.8±10.5	
	**RES+CUR 6.25**	1.5±1.0		33.2±8.2		23.3±3.7		41.9±4.8	
	**RES+CUR 12.5**	1.5±0.1		28.6±11.8	<0.05^2^	25.4±5.1		42.2±13.6	
	**RES+CUR 25**	28.1±8.3	<0.001^3^	36.9±5.3	<0.05^3^	16.6±5.1		18.3±8.9	<0.01^3^

**SCC-15**		**SUB-G1**		**G0/G1**		**S**		**G2/M**	
		**Mean±SD**	**p**	**Mean±SD**	**p**	**Mean±SD**	**p**	**Mean±SD**	**p**
	**DMSO**	2.5±0.9		44.5±9.7		22.3±1.7		31.0±8.6	
	**RES 6.25**	1.9±0.7		43.9±9.4		23.6±2.4		31.8±8.3	
	**RES 12.5**	3.5±1		25.7±9.9		25.0±2.4		46.7±11.3	
	**RES 25**	5.7±0.4		17.2±5.5		32.5±3.6		45.5±8.6	
	**CUR 6.25**	2.1±0.6		51.1±7.3		19.5±2.4		28.3±6.2	
	**CUR 12.5**	16.4.±8.1		26.3±6.8		24.8±9.2		33.5±9.8	
	**CUR 25**	30.0±7.6		26.2±1.6		29.9±7.7		19.5±6.8	
	**RES+CUR 6.25**	2.6±0.5		36.1±6.3	<0.05^1^	25.2±1.3		37.5±6.7	
	**RES+CUR 12.5**	11.4±2.6		30.0±3.5		29.2±4.4		30.9±7.1	
	**RES+CUR 25**	58.2±8.7	<0.001^3^	23.7±3.2		12.7±4.0	<0.001^3^	7.0±2.5	

**FaDu**		**SUB-G1**		**G0/G1**		**S**		**G2/M**	
		**Mean±SD**	**p**	**Mean±SD**	**p**	**Mean±SD**	**p**	**Mean±SD**	**p**
	**DMSO**	2.5±0.6		50.4±3.7		16.3±2.7		30.8±1.0	
	**RES 6.25**	1.3±0.3		23.6±5.6		26.9±3.2		48.2±4	
	**RES 12.5**	1.9±0.5		15.2±5.1		28.5±4		54.3±7.7	
	**RES 25**	4.8±1.4		7.1±0.4		19.9±4.6		68.2±3	
	**CUR 6.25**	1.2±0.6		35.3±7.4		22.3±3.7		41.1±4.5	
	**CUR 12.5**	2.5.±1.3		30.2±6.9		19.1±2.3		48.2±7.9	
	**CUR 25**	9±2.7		25.2±2		16.6±3.1		49.1±5.9	
	**RES+CUR 6.25**	1.6±0.4		16.5±4.9	<0.01^1^	28.5±1		53.3±5.9	
	**RES+CUR 12.5**	3.2±1.4		10.9±4.4	<0.01^2^	23.4±2.8		62.5±7.8	
	**RES+CUR 25**	24.9±6.1	< 0.001^3^	40.4±4.2	<0.01^3^	14.2±1		20.6±5.4	< 0.001^3^

1 Percentage of cells in the sub-G1, G0/G1, S and G2/M phase were calculated using Cell Quest software. The data are representative of three experiments. RES and CUR were used in the range 6.25-25 μM. Statistical significance of the effects obtained with combined treatment was calculated vs. those obtained with the more potent single compound (^1^RES+CUR 6.25 μM vs CUR 6.25 μM; ^2^RES+CUR 12.5 μM vs CUR 12.5 μM; ^3^RES+CUR 25 μM vs CUR and vs RES 25 μM).

**Figure 2:**
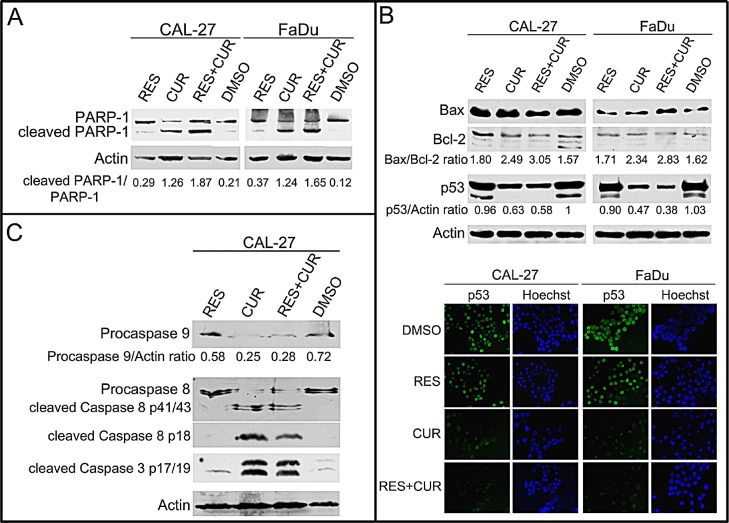
Effect of RES and CUR alone or in combination on apoptosis Panel A: Cleavage of PARP-1 in RES-and CUR-treated HNSCC lines. Western blotting was performed on cells treated with a concentration of 25 μM of the drugs or the DMSO vehicle for 24 h. Actin was used as an internal control. The intensities of the bands obtained in two independent experiments were quantified using ImageJ software after blot scanning, and the densitometric ratios between the cleaved and the full length PARP-1 are reported. Panel B: Assessment of Bax, Bcl-2 and p53 levels with Western blotting in CAL-27 and FaDu cells treated for 48 hours with RES and CUR alone or in combination at 25 μM or with DMSO as vehicle. Sub-cellular localization of p53 was analyzed by indirect immunofluorescence. After treatment, cells were fixed and incubated with anti-p53 antibody. After two washes with PBS, cells were incubated with the secondary Alexa fluor-488-conjugated goat anti-mouse IgG antibody. Nuclei were stained with Hoechst 33342. Original magnification x400. Panel C: CAL-27 cells were treated with compounds, and the expression of procaspases (9-8) and caspases (8-3) was analyzed with Western blotting. The intensities of the bands obtained in two independent experiments were quantified using ImageJ software after blot scanning, and the densitometric ratios between procaspase 9 and actin are reported.

To corroborate that the effect of compounds on the increase of cells in sub-G1 was due to the induction of apoptosis, the cleavage of poly (ADP-ribose) polymerase-1 (PARP-1) was analyzed by Western blotting in CAL-27 and FaDu cell lines treated with 25 μM RES, CUR, RES+CUR or DMSO for 24 hours (Figure 2, Panel A). CUR treatment resulted in considerable PARP-1 proteolytic cleavage (CAL-27: p<0.01 vs control or RES; FaDu: p<0.001 vs control and p<0.01 vs RES) (Figure 2, Panel A). Moreover, RES+CUR treatment increased PARP-1 proteolytic cleavage compared to CUR treatment in both cell lines (CAL-27: p<0.05; FaDu: p<0.01).

To further analyze the activation of apoptosis, treated CAL-27 and FaDu cells were analyzed for the expression of Bax and Bcl-2 with Western blotting. CUR treatment increased the Bax/Bcl-2 ratio in cell lines compared to DMSO treatment (p<0.01) (Figure 2, Panel B). It is of note that the decreased ratio is mainly due to Bcl-2 down-regulation. RES+CUR treatment was more effective in increasing the Bax/Bcl-2 ratio than the CUR treatment (CAL-27: p<0.01; FaDu: p<0.05) (Figure 2, Panel B).

Next, p53 expression was analyzed. Two p53 products were detected in both DMSO- and RES-treated cells. The presence of a truncated p53 protein in FaDu cells was previously reported [27]. Although RES did not affect p53 expression, CUR and RES+CUR reduced the expression of the higher molecular weight p53 protein compared to DMSO (CAL-27: p<0.05; FaDu: p<0.001). The lower molecular p53 protein was not detected after CUR and RES+CUR treatments (Figure 2, Panel B). In addition, nuclear p53 expression was down-regulated in CUR- and RES+CUR-treated cells compared to DMSO-treated cells (Figure 2, Panel B).

The expression of procaspases (9-8) and caspases (8-3) was analyzed with Western blotting in CAL-27 cells to determine which apoptotic pathway was activated after treatments. CUR reduced the level of procaspase 9 and 8 compared to DMSO (p<0.001), thus indicating their activation [28] (Figure 2, Panel C). In addition, procaspase 8 and 3 were cleaved after CUR treatment. RES did not activate caspases. RES+CUR activated caspases similarly to CUR (Figure 2, Panel C).

### Effect of RES and CUR alone or in combination on pro-survival signaling proteins

**Figure 3:**
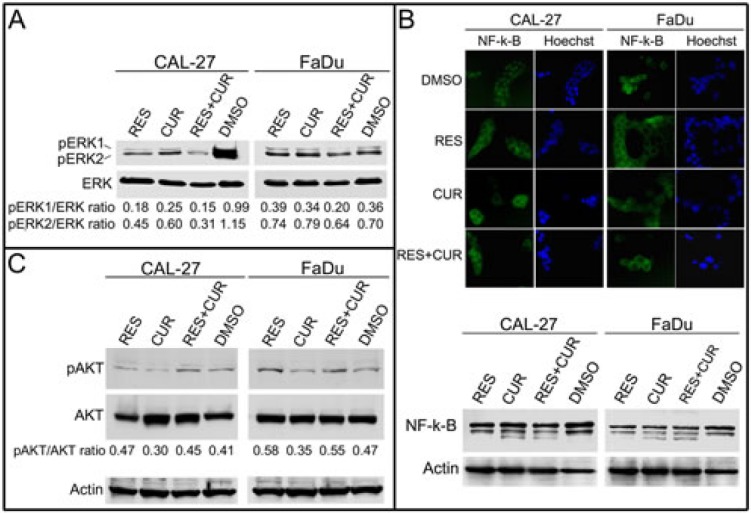
Effect of RES and CUR alone or in combination on pro-survival signaling proteins Panel A: ERK1/ERK2 phosphorylation status in treated HNSCC lines. Western blotting was performed on cells treated with RES and CUR alone or in combination at 25 μM or cells treated with the DMSO vehicle for 48 h. The levels of phosphorylated ERK1/ERK2 were compared with those of the total ERK proteins, and the ratios are reported. Panel B: Inhibition of nuclear translocation of NF-κB after treatment with RES, CUR, or RES+CUR in HNSCC cells. After treatment, the cells were fixed and incubated with anti-NF-κB antibody. After two washes with PBS, the cells were incubated with the secondary Alexa fluor-488-conjugated goat anti-mouse IgG antibody. Nuclei were stained with Hoechst. Western blotting was performed on cells treated with RES and CUR alone or in combination at 25 μM or with the DMSO vehicle for 48 h. Representative experiments are shown. Panel C: AKT phosphorylation status in treated HNSCC lines. Western blotting was performed on cells treated with RES and CUR alone or in combination at 25 μM or cells treated with the DMSO vehicle for 48 h. The level of phosphorylated AKT (pAKT) was compared with that of total AKT, and the ratios are reported. Actin was used as control of loading.

The effect of the compounds on ERK phosphorylation (p-ERK) in unstimulated CAL-27 and FaDu cells was then investigated. RES and CUR reduced p-ERK1 and p-ERK2 levels in CAL-27 cells (p-ERK1: p<0.001; p-ERK2: p<0.01). When administered alone, neither RES nor CUR had notable effects on p-ERK levels in FaDu cells. However, RES potentiated the inhibition of p-ERK1 by CUR in both cell lines (p<0.01). In addition, RES+CUR further reduced p-ERK2 compared to RES or CUR in both cell lines (p<0.05) (Figure 3, panel A).

NF-κB is implicated in the survival and invasiveness of HNSCC cells, and different chemotherapeutic compounds inhibit NF-κB activation [29-31]. The activation of NK-κB leads to its translocation from the cytoplasm to the nucleus. To determine whether RES, CUR and RES+CUR treatments were able to interfere with NK-κB nuclear translocation, CAL-27 and FADU cells were treated with compounds alone or in combination at a concentration of 25 μM for 24 hours. NF-κB was found to be mainly localized in the nuclei in DMSO-treated cells (Figure 3, Panel B). Conversely, RES and CUR alone or in combination induced NF-κB accumulation in the cytoplasm in both cell lines (Figure 3, Panel B). In addition, Western blotting analysis demonstrated the appearance of an extra, lower molecular weight product of NF-κB in CUR- and RES+CUR-treated cells but not in DMSO and RES-treated cells. This smaller molecular protein may represent an NF-κB degradation product (Figure 3, Panel B). The effect of the compounds on AKT phosphorylation in unstimulated CAL-27 and FaDu cells was then investigated (Figure 3, Panel C). RES alone significantly potentiated AKT phosphorylation in CAL-27 and FaDu cell lines compared to DMSO treated cells (p<0.05). Conversely, CUR decreased the level of phosphorylated AKT (pAKT) both in CAL-27 and FaDu cells (p<0.01 and p<0.05 respectively). However, treatment with RES+CUR increased the level of pAKT in FaDu cells compared to DMSO (p<0.05).

### Induction of autophagy in HNSCC cells after treatment with RES, CUR, or RES + CUR

**Figure 4:**
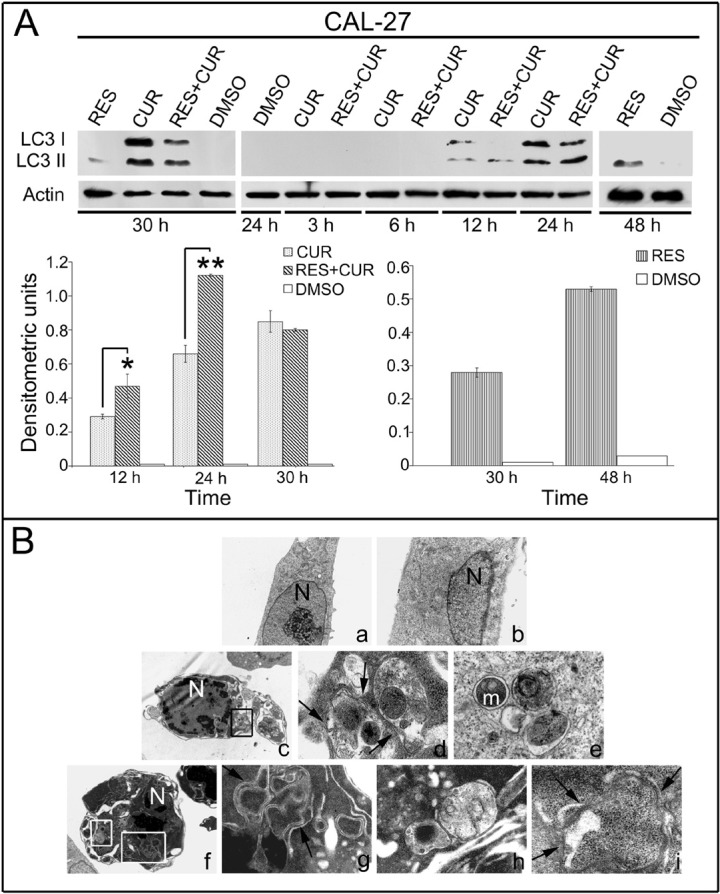
Analysis of autophagy in HNSCC treated cells Panel A: Expression and densitometric quantization of LC3 II HNSCC cells after treatment with RES, CUR, or RES+CUR using Western blotting (*p<0.05, **p<0.01). Panel B: Ultrastructural analysis of autophagy in HNSCC cells by transmission electron microscopy. a. DMSO-treated cells; b. RES-treated cells; c-e. CUR-treated cells; f-i. RES+CUR-treated cells. d. Higher magnification of the square in c; g. Higher magnification of the bigger square in f; h. Higher magnification of the smaller square in f. Arrows indicate double membrane vacuoles.

To determine the effect of the treatments in inducing autophagy in HNSCC cells, the expression pattern of the autophagosomal marker microtubule-associated protein 1 light chain 3 (LC3) was analyzed with Western blotting (Figure 4, Panel A). During autophagy, ProLC3 is processed to its LC3 I cytosolic form, which is subsequently modified to a membrane-bound form (LC3 II) that localizes to pre-autophagosomes and autophagosomes, making this protein an autophagosomal marker [32]. LC3 I and LC3 II expression increased after 30 hours of CUR or RES+CUR treatments at the concentration of 12.5 μM compared to DMSO (Figure 4, Panel A). RES induced a slight increase of LC3 II compared to DMSO. LC3 II expression was then analyzed after 3, 6, 12 and 24 hours of treatment. LC3 II was more highly expressed in cells treated with RES+CUR compared to those treated with CUR after 12 and 24 hours of treatment (p<0.05 and p<0.01, respectively). RES-treated cells showed a high expression of LC3 II only after 48 hours of treatment (Figure 4, panel A).

Induction of autophagy was confirmed using transmission electron microscopy [33]. Cells were treated with different compounds at the concentration of 12.5 μM for 24 hours. No difference was found between RES- or DMSO-treated cells (Figure 4, Panel B). Conversely, CUR-treated cells showed the presence of cytoplasmatic autophagic vacuoles surrounded by double membrane and containing organelles such as mitochondria. Apoptotic cells were also revealed after CUR treatment. Notably, the morphological features of apoptosis and autophagy were simultaneously observed in the same cells. RES+CUR-treated cells were similar to CUR-treated cells. In addition, combined treatment more actively stimulated the formation of double membranes surrounding vast portions of cytoplasm. This phenomenon was most evident in cells that already showed the morphological characteristic of apoptosis (Figure 4, Panel B).

### CUR induces Reactive Oxygen Species (ROS) production in human HNSCC cells and SALTO mouse salivary gland cancer cells

To determine the effect of the compounds alone or in combination on intracellular ROS production in human HNSCC and SALTO cells, the DCF-DA assay was performed. The effects of the compounds were compared to each other and to DMSO and the results were expressed as the fluorescence intensity (Table 2). CUR induced a significant dose-dependent ROS production at the concentration of 25 and 50 μM compared to DMSO in all HNSCC cells (p<0.001 for CAL-27 and SCC-15; p<0.01 for FaDu). Conversely, RES did not affect the levels of intracellular ROS in HNSCC cells. RES+CUR was able to significant increase ROS production compared to CUR only in FaDu cells at 50 μM (p<0.001). RES and CUR alone or in combination induced a significant dose-dependent ROS production compared to DMSO in SALTO cells (Table 2).

**Table 2:** Effects of RES and CUR alone or in combination on the intracellular ROS production in human HNSCC cells and SALTO mouse salivary gland cancer cells

	CAL-27		SCC-15		FaDu		SALTO	
	Mean±SD^1^	p	Mean±SD	p	Mean±SD	p	Mean±SD	p
**DMSO**	3398±174		4366±61		3159±19		3358±58	
**RES 6.25**	3328±88		4174±32		3062±5		3473±115	
**RES 12.5**	3338±136		4178±122		3150±49		3524±111	
**RES 25**	3667±233		4333±13		3248±11		3723±28	<0.05^1^
**RES 50**	3409±42		4227±33		3303±44		3783±10	<0.05^1^
**CUR 6.25**	3354±174		3996±50		3311±86		3416±254	
**CUR 12.5**	3387±249		4092±48		3360±157		3705±179	<0.05^1^
**CUR 25**	4497±214	<0.001^1^	5761±151	<0.001^1^	3496±134	<0.01^1^	4222±27	<0.001^1^
**CUR 50**	4933±24	<0.001^1^	5953±352	<0.001^1^	3540±5	<0.01^1^	4286±13	<0.001^1^
**RES+CUR 6.25**	3387±55		3971±47		3263±25		3431±119	
**RES+CUR 12.5**	3329±106		4224±10		3136±45		3460±115	
**RES+CUR 25**	4440±195	<0.001^1^	5864±361	<0.001^1^	3325±36		4351±34	<0.001^1^
**RES+CUR 50**	4905±77	<0.001^1^	5837±304	<0.001^1^	3869±93	<0.001^1,2^	4539±37	<0.001^1^

The results are reported as the mean of the fluorescence intensity ± SD values from three experiments performed in triplicate. RES and CUR were used in the range 6.25-50 μM. Statistical significance of the effects obtained with treatment alone or in combination was calculated vs. those obtained with DMSO and the more potent single compound (^1^RES and CUR alone or in combination **vs** DMSO; ^2^RES+CUR **vs** CUR).

### RES potentiates the apoptotic effect of CUR on SALTO mouse salivary gland cancer cells

**Figure 5:**
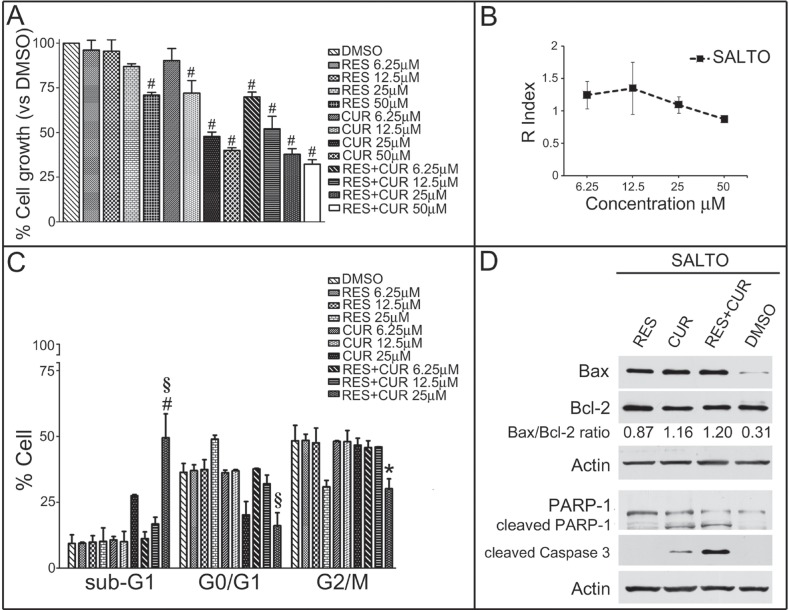
Effect of RES and CUR alone or in combination on proliferation, cell cycle and apoptosis of SALTO cells Panel A: Survival of SALTO cancer cells was assessed using the SRB assay after 48 hours of treatment with DMSO, RES or CUR alone or in equimolar combinations of the two compounds (RES+CUR). The results reported are the mean ± SD values from three experiments performed in triplicate. #: p< 0.001 vs. cultures treated with DMSO. Panel B: Interaction between RES and CUR on the growth of SALTO tumor cells. The graph represents the KERN index (R) after treatment. R > 1 represents a synergistic effect, and R < 1 indicates that the effect of the combined treatment is less than additive. R=1 indicates that the effect is additive. Panel C: Effects of RES and CUR alone or in combination on SALTO cell cycle. Percentage of cells in the sub-G1, G0/G1, and G2/M phases were calculated using Cell Quest software. The data are representative of the three experiments. RES and CUR were used in the range 6.25-25 μM. Statistical significance of the effects obtained with combined treatment was calculated vs** those obtained with the more potent single compound (# p<0.001 RES+CUR 25 μM vs CUR 25 μM; § p<0.001 RES+CUR 25 μM vs RES 25 μM; * p<0.01 RES+CUR 25 μM vs CUR 25 μM). Panel D: Expression of Bax and Bcl-2 levels, cleavage of PARP-1 and expression of proteolytic caspase 3 peptides were assayed with Western blotting in SALTO cells treated for 48 hours with RES and CUR alone or in combination at 25 μM or with DMSO as vehicle control. The intensities of the bands obtained in two independent experiments were quantified using ImageJ software after blot scanning, and the densitometric ratios between the Bax and Bcl-2 are reported.

Survival of SALTO cancer cells was evaluated by the SRB assay after exposure to compounds for 48 hours. The effects of CUR and RES were dose-dependent. RES gained statistical significance at 50 μM compared to the vehicle control treatment (p<0.001). Conversely, CUR treatment was effective at 50-12.5 μM (p<0.001). In addition, the effect obtained on SALTO cells with equimolar combinations of RES+CUR was significant compared to treatment with either RES or CUR alone at concentrations of 50-6.25 μM (p<0.001) and 12.5-6.25 μM (p<0.01), respectively (Figure 5, Panel A).

The interaction between RES+CUR at the concentration of 50 μM produced an R index of 0.88, which indicates that at that concentration, the effect of the association of the two compounds was less than additive. Although R was 1.1, 1.35 and 1.24 at the concentrations of 25, 12.5 and 6.25 μM, respectively, this value was not significantly different compared to R=1, which confirms the additive effect of the compounds (Figure 5, Panel B).

The IC50 values of CUR or RES+CUR were 29.05 and 15.52, respectively. Thus, the combination treatment of RES + CUR significantly reduces IC50 compared to treatment with CUR alone (p<0.01).

The effects of the compounds alone or in combination on apoptosis and the cell cycle distribution of SALTO cells were also determined (Figure 5, Panel C). RES resulted in a dose-dependent increase in the percentage of G0/G1 cells (p<0.05 vs DMSO at 25 μM) and in an increased percentage of G2/M phase cells (p<0.01 vs DMSO at 25 μM). RES did not change the apoptotic sub-G1 population. Conversely, CUR resulted in a dose dependent increase of the percentage cells in the sub-G1 phase (p<0.01 vs DMSO at 25 μM) and a decrease in the number of G0/G1 cells (p<0.01 vs DMSO at 25 μM). As for the effects of combined treatments, RES+CUR induced a dose-dependent increase in the percentage of apoptotic, sub-G1 cells and a dose-dependent decrease in the percentage of G0/G1 and G2/M cells compared to either compound administered alone at the higher dose (Figure 5, Panel C). From the comparison of the apoptotic rates obtained with the RES+CUR and those obtained with CUR alone, it emerged that the combined treatment allowed a reduction in the dose of CUR required to achieve an apoptotic rate of 49.5% by 1.8 times.

RES and CUR increased the Bax/Bcl-2 ratio in SALTO cells compared to DMSO (p<0.01) (Figure 5, Panel D). CUR but not RES induced PARP-1 and procaspase-3 proteolytic cleavage. However, RES+CUR increased procaspase-3 cleavage compared to CUR (Figure 5, Panel D).

### Delay of tumor growth *in vivo* by treatment with RES and CUR alone or in combination

To evaluate whether the administration of RES and CUR alone or in combination was able to inhibit the growth of transplanted SALTO cells, groups of BALB/c mice were treated with RES and CUR alone or in combination prior to or simultaneously with tumor cell implantation [34]. Corn oil, used as vehicle, and water were used as negative controls.

**Figure 6:**
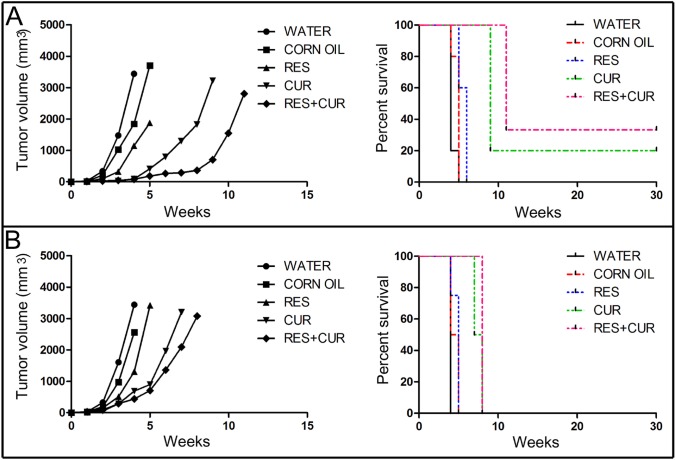
Delay of *in vivo* tumor growth by treatment with RES and CUR alone or in combination Groups of BALB/c mice were treated with RES and CUR alone or in combination prior to (Panel A) or simultaneously with (Panel B) SALTO tumor cell implantation. Differences in the tumor volumes and the mean survival time among the treated mice are reported.

Four weeks following the challenge, CUR and RES+CUR each induced a more efficient tumor mean volume decrease than RES when treatment was started before the tumor challenge (90 mm^3^ and 80 mm^3^ vs 1148 mm^3^, respectively) (p<0.0001). All water-, corn oil- and RES-treated mice were sacrificed for exceeding tumor volume endpoints six weeks after the challenge (Figure 6, Panel A). Conversely, all CUR- and RES+CUR-treated mice remained alive at this time. However, eight weeks after the tumor challenge, the RES+CUR treatment reduced the tumor mean volume more efficiently than the CUR treatment (366 mm^3^ vs 1828 mm^3^) (p=0.0092). Intriguingly, treatment with RES+CUR resulted in a complete regression of tumor growth in 2 out of 6 mice. These mice remained tumor-free until the 30^th^ week. Conversely, only one mouse remained alive until the 30^th^ week in the CUR-treated mice group. When treatment started simultaneously with the tumor challenge, CUR and RES+CUR reduced the mean tumor volume more efficiently than RES (691 mm^3^ and 440 mm^3^ vs 1311 mm^3^, respectively) (p<0.05) four weeks following the challenge. All water-, corn oil- and RES-treated mice were sacrificed for exceeding tumor volume endpoints within 5 weeks, and the CUR- and RES+CUR-treated mice were sacrificed by the 9^th^ week (Figure 6, Panel B). Of note, RES+CUR reduced the mean tumor volume more efficiently than CUR alone (2092 mm^3^ vs 3203 mm^3^) (p=0.046) after seven weeks.

RES prolonged median survival time compared to water (6 vs 4 weeks) (p=0.009) and corn oil (6 vs 5 weeks) (p=0.041), but CUR prolonged median survival time compared to RES when treatment started before the tumor challenge. However, it is important to note that, although the RES+CUR treatment prolonged median survival time when compared to CUR treatment (11 vs 9 weeks), the delay was not significant. When treatment with compounds started with the tumor challenge, the median survival time of RES+CUR-treated mice versus the survival of water-, corn oil- or RES-treated mice was 8 vs 4 weeks (p=0.008), 8 vs 4.5 weeks (p=0.0084) and 8 vs 5 weeks (p=0.01), respectively.

Overall, the risk of developing tumors in water- and corn oil-treated mice was 15.29 and 9.74 times greater than in the RES-treated mice, 33.69 and 23.08 times greater than in the CUR-treated mice and 41.31 and 27.58 times greater than in the RES+CUR-treated mice when the treatments started before the tumor challenge (Table 3). Moreover, the risk of developing tumors in the RES-treated mice was 20.84 and 26.22 time greater than in the CUR- and RES+CUR treated mice, respectively. No difference in the risk of tumor development was found between the CUR- and RES+CUR-treated mice. When treatments started with the tumor challenge, the risk of developing tumors in the water-treated mice was 16.44 times greater than in the RES-treated mice and 33.12 times greater than in the CUR- and RES+CUR-treated mice (Table 3). The risk in the corn oil-treated mice was 19.60 times greater than in the CUR- and RES+CUR-treated mice (Table 3). Finally, the risk of developing tumors in the RES-treated mice was 19.95 times greater than in the CUR- and RES+CUR treated mice groups. No difference was found between the CUR- and RES+CUR-treated mice (Table 3).

**Table 3:** Comparison of mice survival by log-rank (Mantel-Cox) test

Variable	Contrast	Hazard ratio	95% hazard ratio confidence limitis		p value	Median survival (weeks)
			lower	upper		
**Treatment started before tumor challenge**	CORN OIL vs WATER	8.67	0.83	91.10	NS	5 vs 4
	RES vs WATER	15.29	1.97	118.4	0.009	6 vs 4
	CUR vs WATER	33.69	3.79	299.2	0.0016	9 vs 4
	RES+CUR vs WATER	41.31	4.72	361.4	0.0008	11 vs 4
	RES vs CORN OIL	9.74	1.09	86.46	0.041	6 vs 5
	CUR vs CORN OIL	23.08	2.81	189.3	0.0035	9 vs 5
	RES+CUR vs CORN OIL	27.58	3.44	220.8	0.0018	11 vs 5
	CUR vs RES	20.84	2.78	156.2	0.0031	9 vs 6
	RES+CUR vs RES	26.22	3.5	196.6	0.0015	11 vs 6
	RES+CUR vs CUR	5.55	0.73	41.99	NS	11 vs 9

**Treatment started after tumor challenge**	CORN OIL vs WATER	10.31	0.52	205.9	NS	4.5 vs 4
	RES vs WATER	16.44	1.13	239.3	0.04	5 vs 4
	CUR vs WATER	33.12	2.48	442.7	0.008	7.5 vs 4
	RES+CUR vs WATER	33.12	2.48	442.7	0.008	8 vs 4
	RES vs CORN OIL	2.54	0.17	37.01	NS	5 vs 4.5
	CUR vs CORN OIL	19.60	2.14	179.3	0.0084	7.5 vs 4.5
	RES+CUR vs CORN OIL	19.60	2.14	179.3	0.0084	8 vs 4.5
	CUR vs RES	19.95	2.04	194.8	0.01	7.5 vs 5
	RES+CUR vs RES	19.95	2.04	194.8	0.01	8 vs 5
	RES+CUR vs CUR	10.31	0.51	205.9	NS	8 vs 7.5

NS= not significant

Our results suggest that RES and CUR alone are able to significantly delay tumor growth and prolong median survival in comparison to water and corn oil and that CUR is more potent than RES. However, RES potentiates the effects of CUR in reducing tumor volume. In addition, our results indicate that treatment with RES and CUR is more potent when administered before a tumor challenge.

### Hematological and clinical chemistry parameters in mice treated with RES and CUR alone or in combination

To determine whether oral administration of RES and CUR had side effects on BALB/c mice, hematological and clinical chemistry parameters were analyzed after treatment (Supplementary Tables 1–5). Clinical analysis was evaluated only in mice in which the treatment was started two weeks before the SALTO tumor challenge, and the analysis was performed before the treatment and after 4 and 8 weeks after the tumor challenge [34]. The values of individual mice appeared highly heterogeneous within each group. Post-treatment measurements performed 4 weeks after the tumor challenge revealed alterations in some clinical parameters in each group of mice. Nonetheless, these alterations were less pronounced in the CUR- and RES+CUR-treated groups than in the water-, corn oil-, or RES-treated groups. The water-, corn oil-, and RES-treated groups displayed a marked decrease in the percentage of lymphocytes (p<0.01) and a marked increase in the percentage of granulocytes (p<0.01). In addition, a marked increase in the total number of white blood cells (p<0.05) as well as the level of LDH (p<0.01) was also observed in these three groups. Indeed, at this stage, the CUR- and RES+CUR-treated groups displayed a slight decrease in the percentage of lymphocytes (p<0.05) and a slight increase in the percentage of granulocytes (p<0.05). In addition, an increase in the level of LDH was also observed in both the CUR- and RES+CUR-treated groups (p<0.05).

It is important to note that 8 weeks after the tumor challenge, the CUR-treated mice displayed a marked decrease in the percentage of lymphocytes (p<0.01) and a marked increase in the percentage of granulocytes (p<0.01). Conversely, the percentages of lymphocytes and granulocytes in the RES+CUR-treated mice fell within the reference values. Collectively, these results demonstrated that RES and CUR alone or in combination did not affect hematological and clinical chemistry parameters and that the alterations observed in the water- and corn oil-treated mice and the CUR-treated mice are likely associated with tumor growth.

## DISCUSSION

Therapeutic methods based on drug mixtures focus on enhancing clinical responses while lowering side effects and the incidence of drug resistance. The benefit of combining multiple components arises from the fact that each agent can have a single target or mechanism of action or that different agents may share the same target or mechanism of action against cancer cells [4, 5, 9, 10]. Thus, the combination treatment could either enhance the number of targets and/or the mechanisms of action or increase the effects on the same target, therefore decreasing the drug concentrations required for efficacy. Polyphenols can be used to inhibit the growth of cancer cells due to their ability to affect the activity of multiple targets involved in carcinogenesis. Epidemiological studies have shown the association between the consumption of fruits and vegetables and the prevention of human diseases, including cancer [5, 6, 35-38]. However, it must be considered that the poor bioavailability of polyphenols will influence the effective dose delivered to tumor cells. The aim of this study was to determine whether the combination of RES+CUR resulted in an enhancement of their *in vitro* and *in vivo* antitumor activities on HNSCC cell lines compared to each of the compounds in isolation.

In this study, we provide evidence that CUR inhibits the proliferation and induces apoptosis of HNSCC cell lines and that the combination of RES and CUR potentiates the apoptotic effect of CUR. The apoptotic rates obtained with the RES+CUR allowed a reduction in the dose of CUR required to achieve the same apoptotic rate in HNSCC cell lines by 1.8 to 2.77 times. The model of interaction between CUR and RES when used in combination in HNSCC lines indicated the onset of an additive effect of the two compounds with respect to the associated single treatment after decrease of their concentrations. RES+CUR compared to CUR alone increased PARP-1 proteolytic cleavage and the Bax/Bcl-2 ratio mainly by decreasing Bcl-2 protein expression in HNSCC cell lines. The treatment with CUR and RES + CUR also induced the activation of the initiator caspases 8 and 9 and the effector caspase 3, which activated both the intrinsic and extrinsic apoptotic pathways. CUR is able to trigger apoptosis through both the extrinsic apoptotic pathway by mimicking the binding with the TNF receptor and the intrinsic apoptotic pathway [20, 39, 40]. However, the CUR and RES+CUR treatments decreased the p53 expression in HNSCC cell lines. Several studies have shown that CUR induces apoptosis by increasing the expression of p53 [41-43]. On the other hand, other studies have shown that CUR can induce apoptosis without altering the levels of p53 [40] or even through down-regulating p53 expression [44-46]. Thus, the effect of CUR may be cell-type specific. The decrease of p53 expression in HNSCC cell lines after CUR treatment might not be sufficient to alter the activation of the intrinsic pathway and/or the extrinsic pathway might enhance a weak activation of the intrinsic pathway. In addition, activation of apoptosis might be due to transcriptionally-independent activities of p53 [47].

HNSCC cell lines overexpress EGFR and ErbB2 receptors, which activate ERK1 and ERK2 [48-50]. It was demonstrated that CUR is able to inhibit EGFR and ErbB2 phosphorylation in breast and colon cancer cell lines and that the combined treatment of RES+CUR increases these effects in colon carcinoma cell lines [51]. When activated, ERK1 and ERK2 are capable of mediating a number of proliferative signals and the transcription of anti-apoptotic genes such as Bcl-2 [52]. Here, we observed that RES potentiated the inhibition of p-ERK1 and p-ERK2 by CUR in HNSCC cell lines. This inhibition could also be responsible for the inhibition of cell proliferation induced by treatment with CUR and RES+CUR. In addition, the decreased Bcl-2 expression in HNSCC cell lines after treatment might also be due to the inhibition of p-ERK1 and p-ERK2.

The NF-κB pathway is often aberrantly activated during the development and progression of HNSCC [31]. Liposome-encapsulated CUR was shown to suppress growth of HNSCC *in vivo* and in xenografts through the inhibition of NF-κB pathway. Here, we observed that RES and CUR alone or in combination induced NF-κB accumulation in the cytoplasm in both cell lines and that CUR induced the appearance of an extra, lower molecular weight form of cytoplasmic NF-κB, which might represent a degradation product and thus indicate CUR-stimulated degradation of NF-κB. Caspase-mediated proteolysis of NF-κB has been previously reported [53, 54]. Thus, CUR could induce inactivation of NF-κB also through the activation of caspases. In addition, when active, ERK1 and ERK2 can stimulate nuclear translocation of the transcription factor NF-κB [55]. Accordingly, inhibition of p-ERK1 and p-ERK2 by RES and CUR might be responsible for inhibiting NF-κB nuclear translocation. Moreover, activation of NF-κB induces the expression of Bcl-2 family genes [56]. Thus, NF-κB inhibition could make the initiation of the apoptotic process through Bcl-2 down-regulation possible. The key role of the Bcl-2 decrease after RES+CUR treatment is also emphasized by its significance in the initiation of autophagy. Indeed, we observed that RES+CUR increases the expression of LC3 II an autophagosomal marker compared to CUR alone. Bcl-2 is able of sequestering Beclin 1, which promotes the formation of the autophagosome and coordinates autophagy [57]. The binding of Bcl-2 with Beclin 1 is proteolyzed by the activation of caspase 3. Thus, the decreased expression of Bcl-2 and the activation of caspase 3 after treatment with RES+CUR might be responsible for the release of Beclin 1 in the cytoplasm and activation of autophagy. It has been demonstrated that CUR is able to simultaneously induce apoptosis and autophagy in human squamous cell carcinoma cells [21]. Indeed, we observed that the combined treatment more actively stimulated the formation of double membranes surrounding vast portions of cytoplasm. This phenomenon was most evident in cells that already showed the morphological characteristic of apoptosis. Although a sustained activation of ERK1 and ERK2 is required for the activation and maintenance of autophagy, our results show that RES+CUR inhibit the phosphorylation of ERK1 and ERK2 simultaneously with the formation of autophagic vacuoles [52]. On the other hand, autophagy might result from the ability of CUR to inhibit the signaling mediated by Akt/mTOR/p70S6K and induction of reactive oxygen species (ROS) production as already demonstrated in different tumors [21, 58]. We observed that CUR rapidly induced the production of ROS in HNSCC cells and inhibited AKT phosphorylation. ROS in turn can activate apoptosis and potentiate autophagy [59].

Finally, taking into account that a limited number of animals studies are available on the anti-cancer effects of polyphenols, we evaluated the *in vivo* effects of single and combined RES and CUR treatment in hampering the growth of transplanted Neu-overexpressing BALB-*neu*T salivary gland cancer cells (SALTO) in BALB/c mice. Initially, we demonstrated that RES potentiates the apoptotic effect of CUR on SALTO cells and that the compounds have an additive effect in inhibiting cell growth. Then, we performed an *in vivo* study. RES and CUR alone or in combination were administered prior to or simultaneously with the SALTO tumor challenge. Regardless of the start time of compound administration, we observed that the RES+CUR treatment reduced the mean tumor volume more efficiently than CUR alone. However, although the RES+CUR treatment prolonged the median survival time when compared to the CUR treatment, the delay was not statistically significant when the compounds were given prior to or simultaneously with tumor challenge. Thus, RES potentiated the *in vivo* anti-tumor effect of CUR in agreement with our *in vitro* observations. In addition, the administration of CUR and RES were safe in BALB/c mice. Indeed, RES and CUR alone or in combination did not significantly affect hematological or clinical chemistry parameters.

Salivary gland carcinomas are head and neck tumors that require typical surgical and adjuvant therapy [60]. Although, conservative surgery with nerve monitoring is the state-of-the-art and adjuvant radio(chemo)therapy increases local tumor control, the overall survival is not automatically enhanced [60]. Thus, the development of novel therapies can supplement the pharmaceutical armamentarium presently used for salivary gland carcinomas treatment. We previously demonstrated that intratumoral delivery of recombinant vaccinia virus encoding for ErbB2/Neu could inhibit the growth of salivary gland carcinoma cells in mice [34]. Now, we report that the combination of two nutraceuticals are able to reduce the growth of salivary gland tumors without side effects in mice.

Taken together, our results indicate that the treatment of HNSCC cells with combinations of CUR and RES can be more effective in inhibiting *in vivo* and *in vitro* cancer cell growth than the treatment with CUR alone. Still, additional studies performed both *in vitro* and *in vivo* will be needed to fully define the therapeutic potential of these compounds.

## MATERIAL AND METHODS

### Reagents

DMSO, transresveratrol (RES), curcumin from *Curcuma Longa* (CUR), Sulforhodamine B (SRB), staurosporine and Hoechst 33342 were purchased from Sigma-Aldrich (Milan, Italy). Rabbit polyclonal anti-Bax and mouse monoclonal anti-Bcl-2 antibodies were obtained from BD Pharmingen (BD Biosciences, San Jose, CA, USA). Antibodies against PARP-1, ERK1/2 (C-14), phospho-ERK (E-4), NF-κB and p53 (DO-1) were obtained from Santa Cruz Biotechnology (CA, USA). Rabbit polyclonal anti-actin was purchased from Sigma-Aldrich (Milan, Italy). The anti-activated caspase 3, anti-caspase 9, anti-caspase 8, anti-AKT and anti-phospho-AKT antibodies were purchased from Cell Signaling Technology (MA, USA). Antibody against LC3 was obtained from Novus Biologicals (Littleton, CO, USA). Goat anti-mouse IgG Alexa fluor-488-conjugated antibody was purchased from Life Technologies™ Molecular Probes (Oregon, USA). The goat anti-mouse or -rabbit IgG peroxidase conjugated secondary antibodies were obtained from Sigma-Aldrich (Milan, Italy).

### Cell lines and treatments

Cell lines derived from HNSCCs of the tongue (CAL-27, SCC-15) or pharynx (FaDu) were maintained in RPMI containing 10% fetal bovine serum, 100 U/ml penicillin, and 100 μg/ml streptomycin. For treatments, cells were incubated for the indicated times in the presence of RES and CUR alone or a combination of the two compounds (dose range 6-50 μM) or vehicle control (DMSO ≤0.1%). Neu-overexpressing salivary gland cancer cells (H-2^d^) (SALTO) were kindly provided by Prof. F. Cavallo (University of Torino) and Prof. PL. Lollini (University of Bologna) and maintained in DMEM containing 20% fetal bovine serum (FBS). SALTO cells were established from salivary carcinoma arising in BALB-*neu*T transgenic male mice hemizygous for the p53^172R-H^ transgene driven by the whey acidic protein promoter [61].

### Sulforhodamine B (SRB) assay

Cells were seeded at 4 × 10^3^ cells/well in 96-well plates and incubated at 37° C to allow cell attachment. After 24 hours, the medium was changed and the cells were treated with RES and CUR alone or in combination or with DMSO and incubated for 48 hours. Cells were then fixed with cold trichloroacetic acid (final concentration 10%) for 1 hour at 4° C. The assay was then performed as previously described [48]. The percentage survival of the cultures treated with the compounds or DMSO was calculated by normalization of their O.D. values to those of the untreated control cultures [48]. The three experiments were performed in triplicate.

### FACS analysis

Asynchronized log-phase growing cells (60% confluent, approximately 2.5 × 10^5^ cells/well in 6-well plates) were treated with RES and CUR alone or in combination or with DMSO in a complete culture medium. After 48 hours, adherent cells and suspended cells were harvested, centrifuged at 1,500 rpm for 10 min and washed twice with cold phosphate buffered saline (PBS). The assay was then performed as previously described [62]. Cells were analyzed with flow cytometry using a FACSCalibur cytometer running CellQuest software.

### Western Blotting

In total, 1 × 10^6^ cells were seeded in 100 mm tissue culture dishes 24 hours prior to the addition of 25 μM of each compound alone or in combination or the vehicle control. After 24 and 48 hours of treatment, the cells were harvested, washed twice with cold PBS and lysed in RIPA buffer as previously described [63]. For immunoblot analysis, 50-80 μg of cell lysates were resolved in 10% SDS-PAGE and then transferred to nitrocellulose membranes. Equal loading and transfer of proteins was verified by Ponceau red staining of the membranes and by analyzing actin expression. The assay was then performed as previously described [63]. Total cell lysates were analyzed for NF-κB expression.

### Fluorescent measurement of Reactive Oxygen Species (ROS)

Dichlorofluorescin diacetate (DCF-DA) was used to detect ROS production in cells. Briefly, 2.5 × 10^5^ cells were seeded into 6-well plates and incubated at 37° C to allow cell attachment before treatment. After two washing with PBS, cells were incubated with 10 μM 2′,7′-Dichlorofluorescin diacetate (Sigma-Aldrich, Milan, Italy) in PBS at 37 °C and 5% CO_2_ in the dark for 30 minutes [64]. After two washing, cells were treated with RES and CUR, alone or in combination, in serum-free medium and incubated at 37 °C and 5% CO_2_ in the dark for different times (15 min-4 hours). Then, adherent cells and suspended cells were harvested, centrifuged at 1,250 rpm for 10 min and seeded in 96-well plate (100 μl per well). Fluorescence intensity was measured after 15 and 30 minutes, 1, and 4 hours using a spectrophotometric plate reader at an excitation wavelength of 495 nm and an emission wavelength of 535 nm. Since the highest level of fluorescence was detected at 30 minutes, and then decreased back to the level of the control after one hour of stimulation (data not shown) this experimental time was chosen for subsequent experiments.

### Immunofluorescence

HNSCC cells were seeded (2.5×10^4^ cells/well) in 8 chambers with permanox (Lab-Tek, IL) in 300 μl of culture medium, grown for 24 h, and treated with RES and CUR alone or in combination or with DMSO in a complete culture medium. After 48 hours, the culture medium was removed, and after two washes with PBS, the cells were fixed with 4% paraformaldehyde at room temperature for 15 min and permeabilized with methanol for 10 min at −20° C [49]. The cells were then incubated at room temperature with primary antibodies for 1 h and, after two more washes, with goat anti-mouse IgG Alexa fluor-488-conjugated secondary antibody for 45 min. Nuclei were counterstained with Hoechst 33342. The slides were then mounted with glycerol, observed with an Olympus BX51 fluorescence microscope and analyzed with the IAS software.

### Transmission electron microscopy

Ultrastructural analyses were performed on CAL-27 cells treated with RES and CUR alone or in combination or with DMSO. After treatment, the cells were fixed in 2.5% glutaraldehyde in PBS pH 7.4, and the samples were processed for transmission electron microscopy following routine procedures [65].

### Treatment of BALB/c mice with RES and CUR alone or in combination

BALB/c mice were subcutaneously injected in the right flank with a 0.2 ml suspension containing 1×10^6^ SALTO cells in phosphate-buffered saline (PBS). Groups of BALB/c mice (5 or 6 mice per group) were treated per os with RES and CUR alone (2 mg in 100 μl of corn oil) or in combination (2 mg of RES in 50 μl of corn oil + 2 mg of CUR in 50 μl of corn oil) or with corn oil (100 μl) or water (100 μl) two weeks prior or simultaneously to the SALTO tumor challenge. When treatment started before the tumor challenge, the mice received daily treatment for two weeks before the tumor challenge and then 3 times a week following the challenge. When treatment started simultaneously to the SALTO tumor challenge, the compounds were administered 3 times a week.

The mice were sacrificed at the first signs of distress. Investigation has been conducted in accordance with the ethical standards and according to the Declaration of Helsinki and according to national and international guidelines. All experiments were approved by the Institutional Animal Care and Use Committee (IACUC) and carried out according to the Italian rules (D.L.vo 116/92; CE. 609/86). A veterinary surgeon was present during the experiments. Animal care, before and after the experiments, was carried out only by trained personnel.

### Analysis of antitumor activity *in vivo*

Tumor growth was monitored weekly until tumor-bearing mice were sacrificed when the tumor exceeded a 20 mm width. Tumors were measured by a caliper in two dimensions, and the volumes were calculated using the formula: width^2^ x length/2 [66].

### Measurement of hematological and clinical chemistry parameters

All blood samples were collected in animals under i.p. anesthesia (20 μl/g.b.w. of 1.2% Avertin-2,2,2-tribromoethanol, 2.4% 2-methyl-2-butanol; Sigma-Aldrich, Italy). For determination of hematological parameters, 20 μl of whole blood was collected in K2EDTA microtainers (Becton, Dickinson and Company, USA) and the samples were analyzed using the commercially available automated cell counter “Simply cell” (BPC BioSed s.r.l., Italy). For cytomorphological examination, each sample from peripheral blood smears was prepared using the differential staining Diff-Quick (Dade SpA, Italy) and analyzed under optical microscopy. For determination of clinical chemistry parameters, blood samples were collected in SST microtainers (Serum Separator Tube; Becton, Dickinson and Company) and centrifuged in a microcentrifuge (5415R model; Eppendorf s.r.l., Italy) at 13,000 rpm for 7 min to separate the serum. Cholesterol (CHOL), triglycerides (TRI), glutamic oxaloacetic transaminase (GOT), glutamic pyruvic transaminase (GPT), blood urea nitrogen (BUN) and lactate dehydrogenase (LDH) were measured using the automatic analyzer Keylab (BPC BioSed s.r.l., Rome, Italy).

### Statistical analysis

The distribution of the cell survival data and the FACS analyses were preliminarily verified using the Kolmogorov-Smirnov test, and the data sets were analyzed by one-way analysis of variance (ANOVA) followed by the Newman-Keuls test. Survival curves and tumor volumes were analyzed using the Kaplan–Meier method and compared using the log-rank test with calculation of the SD according to the method of Greenwood. Differences were regarded to be significant when the p value was ≤ 0.05.

## SUPPLEMENTARY TABLES


