# Supplementary material for: Resveratrol potentiates the in vitro and in vivo anti-tumoral effects of curcumin in head and neck carcinomas
Source: Oncotarget. 2014 Sep 26;5(21):10745–62. doi: 10.18632/oncotarget.2534 (PMC4279407; doi:10.18632/oncotarget.2534)
Supplement: Supplementary file 1 [file oncotarget-05-10745-s001.pdf]

Resveratrol potentiates the *in vitro* and *in vivo* anti-tumoral effects of curcumin in head and neck carcinomas

Supplementary Material

Supplementary Table 1: Effect of water in BALB/c mice: complete blood count (CBC) and clinical chemistry parameters.

CBC	Reference values	Mean ± SD pre-treatment	Mean ± SD 4 weeks post- injection
RBC ($10^6/mm^3$)	6.0-12.0	10.9 ± 0.5	11.2 ± 1.1
MCV (μm^3)	43.0-58.0	45.8 ± 0.6	46 ± 0.5
HCT (%)	35.0-60.0	50.1 ± 1.9	51.7 ± 4.7
PLT ($10^3/mm^3$)	250-1200	956.3 ± 57.7	1106.7 ± 187.6
MPV (μm^3)	4.0-7.0	6 ± 0.3	5.8 ± 0.1
WBC ($10^3/mm^3$)	5.0-12.0	9.4 ± 0.3	22.1 ± 2.9
HGB (g/dl)	9.0-19.0	16 ± 0.7	15.8 ± 1.6
MCH (Pg)	15.0-19.0	14.7 ± 0.1	14.1 ± 1.1
MCHC (g/dl)	34.0-39.0	32 ± 0.4	30.6 ± 2.1
RDW (%)	0-99.9	14.8 ± 0.4	16.5 ± 1
LYM (%)	50.0-75.0	54.5 ± 7	11.8 ± 5.8
MID (%)	5.0-15.0	7.8 ± 1.6	5.7 ± 0.6
GRAN (%)	15.0-40.0	37.8 ± 7.2	84.2 ± 8.7
LYM ($10^3/mm^3$)	4.0-10.0	5.2 ± 0.9	2.3 ± 1.9
MID ($10^3/mm^3$)	0-2.0	0.7 ± 0.2	0.9 ± 0.7
GRAN ($10^3/mm^3$)	0.5-6.0	3.5 ± 0.6	18.3 ± 2.5
PCT (%)	0-99.9	0.6 ± 0	0.6 ± 0.1
PDW (%)	0-99.9	9 ± 0.5	8.6 ± 0.2
**Clinical Chemistry**			
CHOL (mg/dl)	50-120	118 ± 46.5	133 ± 17.1
TRI (mg/dl)	80-200	158 ± 79.5	205 ± 6.2
GOT (U/l)	20-250	125 ± 102.2	146 ± 93.1
GPT (U/l)	20-100	30.7 ± 3.8	30.2 ± 0.5
BUN (mg/dl)	10-40	22.7 ± 4	38 ± 1.7
LDH (U/l)	300-900	897 ± 315,2	2283.3 ± 367.4

Abbreviations: SD: Standard Deviation; RBC: Red Blood Cells; MCV: Mean Corpuscular Volume; HCT: Hematocrit; PLT: Platelets; MPV: Mean Platelet Volume; WBC: White Blood Cells; HGB: Hemoglobin; MCH: Mean Corpuscular Hemoglobin; MCHC: Mean Corpuscular Hemoglobin Concentration; RDW: Red Cell Distribution Width; LYM: Lymphocytes; MID: Minimum Inhibitory Dilution; GRAN: Granulocytes; PCT: Plateletcrit; PDW: Platelet Distribution Width; CHOL: cholesterol; TRI: triglycerides; GOT: serum Glutamic Oxaloacetic Transaminase; GPT: serum Glutamic Pyruvic Transaminase; BUN: Blood Urea Nitrogen; LDH: Lactate Dehydrogenase.

Supplementary Table 2: Effect of corn oil in BALB/c mice: complete blood count (CBC) and clinical chemistry parameters.

**CBC**	**Reference values**	**Mean ± SD pre-treatment**	**Mean ± SD 4 weeks post- injection**
RBC ($10^6/mm^3$)	6.0-12.0	10.8 ± 1.9	9.9 ± 1.1
MCV (μm^3)	43.0-58.0	49.8 ± 1.2	51.1 ± 0.6
HCT (%)	35.0-60.0	51.7 ± 7.7	51.0 ± 5.2
PLT ($10^3/mm^3$)	250-1200	521.3 ± 207.5	605 ± 312.8
MPV (μm^3)	4.0-7.0	6.5 ± 0.5	6.4 ± 0.3
WBC ($10^3/mm^3$)	5.0-12.0	10.1 ± 1.6	28.6 ± 8.9
HGB (g/dl)	9.0-19.0	16.4 ± 0.6	14.6 ± 1.9
MCH (Pg)	15.0-19.0	12.9 ± 0.4	14.6 ± 0.3
MCHC (g/dl)	34.0-39.0	26.5 ± 1.2	28.6 ± 1
RDW (%)	0-99.9	24.3 ± 0.8	16.2 ± 0.8
LYM (%)	50.0-75.0	61.1 ± 5.6	26.6 ± 7.5
MID (%)	5.0-15.0	10.2 ± 1.3	11.9 ± 5.2
GRAN (%)	15.0-40.0	23.0 ± 4.4	61.5 ± 12.4
LYM ($10^3/mm^3$)	4.0-10.0	5.7 ± 1.5	7.3 ± 4
MID ($10^3/mm^3$)	0-2.0	1 ± 0.3	3.2 ± 2.8
GRAN ($10^3/mm^3$)	0.5-6.0	2.2 ± 0.8	18.1 ± 4.1
PCT (%)	0-99.9	0.7 ± 0.3	0.4 ± 0.2
PDW (%)	0-99.9	9.8 ± 1	9.6 ± 0.4
**Clinical Chemistry**			
CHOL (mg/dl)	50-120	162 ± 7	173.3 ± 14
TRI (mg/dl)	80-200	88.3 ± 6.7	100 ± 4
GOT (U/l)	20-250	146 ± 76.9	180 ± 111.1
GPT (U/l)	20-100	27.3 ± 7	24.9 ± 0.9
BUN (mg/dl)	10-40	32 ± 2	54.7 ± 4.6
LDH (U/l)	300-900	768.7 ± 165.2	3872.0± 1155.5

Abbreviations: SD: Standard Deviation; RBC: Red Blood Cells; MCV: Mean Corpuscular Volume; HCT: Hematocrit; PLT: Platelets; MPV: Mean Platelet Volume; WBC: White Blood Cells; HGB: Hemoglobin; MCH: Mean Corpuscular Hemoglobin; MCHC: Mean Corpuscular Hemoglobin Concentration; RDW: Red Cell Distribution Width; LYM: Lymphocytes; MID: Minimum Inhibitory Dilution; GRAN: Granulocytes; PCT: Plateletcrit; PDW: Platelet Distribution Width; CHOL: cholesterol; TRI: triglycerides; GOT: serum Glutamic Oxaloacetic Transaminase; GPT: serum Glutamic Pyruvic Transaminase; BUN: Blood Urea Nitrogen; LDH: Lactate Dehydrogenase.

Supplementary Table 3: Lack of side effects of RES in BALB/c mice: complete blood count (CBC) and clinical chemistry parameters.

**CBC**	**Reference values**	**Mean ± SD pre-treatment**	**Mean ± SD 4 weeks post- injection**
RBC ($10^6/mm^3$)	6.0-12.0	9.8 ± 1	11.3 ± 1.1
MCV (μm^3)	43.0-58.0	49.6 ± 1.1	51.8 ± 0.9
HCT (%)	35.0-60.0	53.2 ± 5.9	58.3 ± 4.7
PLT ($10^3/mm^3$)	250-1200	733.3 ± 76.4	1023.7 ± 190.5
MPV (μm^3)	4.0-7.0	6.5 ± 0.5	6.9 ± 0.4
WBC ($10^3/mm^3$)	5.0-12.0	9.9 ± 1.3	42.2 ± 11.8
HGB (g/dl)	9.0-19.0	15.8 ± 0.8	16.7 ± 1.6
MCH (Pg)	15.0-19.0	13.3 ± 0.6	14.7 ± 0.3
MCHC (g/dl)	34.0-39.0	26.7 ± 1.9	28.5 ± 1
RDW (%)	0-99.9	19.8 ± 4.3	16.6 ± 0.4
LYM (%)	50.0-75.0	52.5 ± 2.5	17.8 ± 7.3
MID (%)	5.0-15.0	9.5 ± 1.3	8.1 ± 0.9
GRAN (%)	15.0-40.0	25.8 ± 5.1	74.2± 8.1
LYM ($10^3/mm^3$)	4.0-10.0	5.3 ± 0.6	5.7 ± 1.6
MID ($10^3/mm^3$)	0-2.0	1 ± 0.3	2.8 ± 1.7
GRAN ($10^3/mm^3$)	0.5-6.0	2.8 ± 1.9	33.7 ± 8.5
PCT (%)	0-99.9	0.7 ± 0.3	0.7 ± 0.1
PDW (%)	0-99.9	10 ± 0	10.5 ± 0.7
**Clinical Chemistry**			
CHOL (mg/dl)	50-120	150 ± 8.7	157.3 ± 10.1
TRI (mg/dl)	80-200	88.7 ± 5.5	128 ± 38.2
GOT (U/l)	20-250	77.7 ± 11.2	104 ± 42.3
GPT (U/l)	20-100	31.0 ± 3.6	34.2 ± 4
BUN (mg/dl)	10-40	31.7 ± 3.5	45.3 ± 4.6
LDH (U/l)	300-900	751.7 ± 47.5	3046.7 ± 732.5

Abbreviations: SD: Standard Deviation; RBC: Red Blood Cells; MCV: Mean Corpuscular Volume; HCT: Hematocrit; PLT: Platelets; MPV: Mean Platelet Volume; WBC: White Blood Cells; HGB: Hemoglobin; MCH: Mean Corpuscular Hemoglobin; MCHC: Mean Corpuscular Hemoglobin Concentration; RDW: Red Cell Distribution Width; LYM: Lymphocytes; MID: Minimum Inhibitory Dilution; GRAN: Granulocytes; PCT: Plateletcrit; PDW: Platelet Distribution Width; CHOL: cholesterol; TRI: triglycerides; GOT: serum Glutamic Oxaloacetic Transaminase; GPT: serum Glutamic Pyruvic Transaminase; BUN: Blood Urea Nitrogen; LDH: Lactate Dehydrogenase.

Supplementary Table 4: Lack of side effects of CUR in BALB/c mice: complete blood count (CBC) and clinical chemistry parameters.

CBC	Reference values	Mean ± SD pre-treatment	Mean ± SD 4 weeks post- injection	Mean ± SD 8 weeks post- injection
RBC ($10^6/mm^3$)	6.0-12.0	10.3 ± 1.5	11.3 ± 0.3	10.6 ± 0.8
MCV (μm^3)	43.0-58.0	49.8 ± 1.6	48.1 ± 2.7	45.9 ± 3.4
HCT (%)	35.0-60.0	52.2 ± 6.3	54.4 ± 4.6	49 ± 7
PLT ($10^3/mm^3$)	250-1200	728.3 ± 84.6	871 ± 108.9	966 ± 109.5
MPV (μm^3)	4.0-7.0	6.5 ± 0.5	6.3 ± 0.7	6 ± 0.4
WBC ($10^3/mm^3$)	5.0-12.0	10.3 ± 2	17.4 ± 4.3	19.3 ± 4.7
HGB (g/dl)	9.0-19.0	15.8 ± 0.8	17 ± 0.4	15.1 ± 1.6
MCH (Pg)	15.0-19.0	13.3 ± 0.6	15.1 ± 0.2	14.2 ± 0.6
MCHC (g/dl)	34.0-39.0	26.7 ± 1.9	31.4 ± 2	31 ± 1.1
RDW (%)	0-99.9	23.5 ± 1.5	16.8 ± 1.6	17.2 ± 0.9
LYM (%)	50.0-75.0	57 ± 2.6	43.7 ± 4.4	23.7 ± 6.9
MID (%)	5.0-15.0	9.5 ± 1.3	5.5 ± 0.9	7.3 ± 1.2
GRAN (%)	15.0-40.0	25 ± 5	50.8 ± 3.5	69 ± 7.9
LYM ($10^3/mm^3$)	4.0-10.0	5.5 ± 0.5	8.4 ± 2.7	4.3 ± 0.7
MID ($10^3/mm^3$)	0-2.0	1.1 ± 0.4	1.2 ± 0.6	1.4 ± 0.3
GRAN ($10^3/mm^3$)	0.5-6.0	2.8 ± 1.3	7.9 ± 6.3	13.6 ± 4.5
PCT (%)	0-99.9	0.5 ± 0.5	0.6 ± 0.1	0.6 ± 0.1
PDW (%)	0-99.9	10 ± 0.5	9.5 ± 1.3	9 ± 0.7
**Clinical Chemistry**				
CHOL (mg/dl)	50-120	151.7 ± 12.6	162.7 ± 28.1	125.3 ± 34.9
TRI (mg/dl)	80-200	125 ± 51.1	161.3 ± 56	116 ± 41.3
GOT (U/l)	20-250	113.3 ± 32.1	134.7 ± 20.1	112 ± 87.2
GPT (U/l)	20-100	42 ± 13.1	49.5 ± 13.9	31.1 ± 16.6
BUN (mg/dl)	10-40	32 ± 2	46.7 ± 12.9	55.3 ± 17.9
LDH (U/l)	300-900	751.7 ± 147.5	2921.3 ± 1169.6	1037.3 ± 273

Abbreviations: SD: Standard Deviation; RBC: Red Blood Cells; MCV: Mean Corpuscular Volume; HCT: Hematocrit; PLT: Platelets; MPV: Mean Platelet Volume; WBC: White Blood Cells; HGB: Hemoglobin; MCH: Mean Corpuscular Hemoglobin; MCHC: Mean Corpuscular Hemoglobin Concentration; RDW: Red Cell Distribution Width; LYM: Lymphocytes; MID: Minimum Inhibitory Dilution; GRAN: Granulocytes; PCT: Plateletcrit; PDW: Platelet Distribution Width; CHOL: cholesterol; TRI: triglycerides; GOT: serum Glutamic Oxaloacetic Transaminase; GPT: serum Glutamic Pyruvic Transaminase; BUN: Blood Urea Nitrogen; LDH: Lactate Dehydrogenase.

Supplementary Table 5: Lack of side effects of RES+CUR in BALB/c mice: complete blood count (CBC) and clinical chemistry parameters.

CBC	Reference values	Mean ± SD pre-treatment	Mean ± SD 4 weeks post- injection	Mean ± SD 8 weeks post- injection
RBC ($10^6/mm^3$)	6.0-12.0	10.5 ± 1.3	10.6 ± 0.5	10.4 ± 0.7
MCV (μm^3)	43.0-58.0	51.6 ± 1.9	53.8 ± 0.2	47.6 ± 2.9
HCT (%)	35.0-60.0	51.5 ± 5.7	57.3 ± 2.9	49.8 ± 2.4
PLT ($10^3/mm^3$)	250-1200	734 ± 75.3	766.3 ± 99.2	716.7 ± 245.4
MPV (μm^3)	4.0-7.0	6.3 ± 0.6	6.1 ± 0.3	6.5 ± 0.3
WBC ($10^3/mm^3$)	5.0-12.0	12.5 ± 1.5	13.9 ± 1	6.5 ± 1.3
HGB (g/dl)	9.0-19.0	16 ± 0.6	16.4 ± 0.9	14.8 ± 0.9
MCH (Pg)	15.0-19.0	14.4 ± 1.3	15.4 ± 0.2	14.2 ± 1
MCHC (g/dl)	34.0-39.0	26.5 ± 1.7	28.6 ± 0.2	29.7 ± 0.6
RDW (%)	0-99.9	20.6 ± 4.2	16.7 ± 0.8	16.2 ± 2.1
LYM (%)	50.0-75.0	51.9 ± 6.2	38.3 ± 5.6	50.5 ± 5.6
MID (%)	5.0-15.0	11.1 ± 2.6	10.1 ± 3.2	11.3 ± 3.2
GRAN (%)	15.0-40.0	32.2 ± 8.5	51.6 ± 8.8	38.2 ± 2.5
LYM ($10^3/mm^3$)	4.0-10.0	5.6 ± 0.7	4.4 ± 1.7	3.3 ± 1
MID ($10^3/mm^3$)	0-2.0	1.4 ± 0.6	1.5 ± 0.4	0.7 ± 0.1
GRAN ($10^3/mm^3$)	0.5-6.0	3.4 ± 2.2	8 ± 2.1	2.4 ± 0.3
PCT (%)	0-99.9	0.3 ± 0.3	0.5 ± 0.1	0.5 ± 0.2
PDW (%)	0-99.9	9.2 ± 0.7	9.1 ± 0.4	9.7 ± 0.4
**Clinical Chemistry**				
CHOL (mg/dl)	50-120	139.3 ± 17.9	137.3 ± 8.3	159.3 ± 23.9
TRI (mg/dl)	80-200	92 ± 7.2	68 ± 28	146.7 ± 70.2
GOT (U/l)	20-250	122 ± 25.5	100 ± 27.7	148 ± 125.8
GPT (U/l)	20-100	31.3 ± 5.8	27 ± 1.7	48.8 ± 26
BUN (mg/dl)	10-40	35.7 ± 4	44 ± 4	53.3 ± 2.3
LDH (U/l)	300-900	996 ± 451.7	1581.3 ± 330.1	910.7 ± 445.5

Abbreviations: SD: Standard Deviation; RBC: Red Blood Cells; MCV: Mean Corpuscular Volume; HCT: Hematocrit; PLT: Platelets; MPV: Mean Platelet Volume; WBC: White Blood Cells; HGB: Hemoglobin; MCH: Mean Corpuscular Hemoglobin; MCHC: Mean Corpuscular Hemoglobin Concentration; RDW: Red Cell Distribution Width; LYM: Lymphocytes; MID: Minimum Inhibitory Dilution; GRAN: Granulocytes; PCT: Plateletcrit; PDW: Platelet Distribution Width; CHOL: cholesterol; TRI: triglycerides; GOT: serum Glutamic Oxaloacetic Transaminase; GPT: serum Glutamic Pyruvic Transaminase; BUN: Blood Urea Nitrogen; LDH: Lactate Dehydrogenase.
